# Metabolic Conditions and Peri-Implantitis

**DOI:** 10.3390/antibiotics12010065

**Published:** 2022-12-29

**Authors:** Asma Gasmi Benahmed, Amin Gasmi, Torsak Tippairote, Pavan Kumar Mujawdiya, Oleksandr Avdeev, Yurii Shanaida, Geir Bjørklund

**Affiliations:** 1 Académie Internationale de Médecine Dentaire Intégrative, 75000 Paris, France; 2 Société Francophone de Nutrithérapie et de Nutrigénétique Appliquée, 69100 Villeurbanne, France; 3 Faculty of Medicine Ramathibodi Hospital and Institute of Nutrition, Mahidol University, Bangkok 10400, Thailand; 4 Nutritional and Environmental Medicine Department, BBH Hospital, Bangkok 10540, Thailand; 5 Birla Institute of Technology and Science-Pilani, Hyderabad 500078, India; 6 Pediatric Dentistry Department, I. Horbachevsky Ternopil National Medical University, 46003 Ternopil, Ukraine; 7 Council for Nutritional and Environmental Medicine, 8610 Mo i Rana, Norway

**Keywords:** dental implants, inflammation, peri-implantitis, metabolic disorders, dental biomaterials, antibiotics

## Abstract

Dental implants to replace lost teeth are a common dentistry practice nowadays. Titanium dental implants display a high success rate and improved safety profile. Nevertheless, there is an increasing peri-implantitis (PI), an inflammatory disease associated with polymicrobial infection that adversely affects the hard and soft tissues around the implant. The present review highlights the contribution of different metabolic conditions to PI. The considerations of both local and systemic metabolic conditions are crucial for planning successful dental implant procedures and during the treatment course of PI. Un- or undertreated PI can lead to permanent jaw bone suffering and dental implant losses. The common mediators of PI are inflammation and oxidative stress, which are also the key mediators of most systemic metabolic disorders. Chronic periodontitis, low-grade tissue inflammation, and increased oxidative stress raise the incidence of PI and the underlying systemic metabolic conditions, such as obesity, diabetes mellitus, or harmful lifestyle factors (cigarette smoking, etc.). Using dental biomaterials with antimicrobial effects could partly solve the problem of pathogenic microbial contamination and local inflammation. With local dentistry considering factors, including oral microbiota and implant quality control, the inclusion of the underlying systemic metabolic conditions into the pre-procedure planning and during the treatment course should improve the chances of successful outcomes.

## 1. Introduction

Dental issues are common health concerns that require immediate attention to relieve excruciating pain and prevent tooth deterioration [1]. There is a close association between oral and dental health, in which maintaining good oral hygiene directly links with better dental health [2]. According to an estimation, around 267 million people around the world are suffering from tooth loss [3]. The permanent tooth loss and alveolar bone defects can be managed by moveable dentures that are fixed in soft tissues and need to be changed over time. Now, dental implants have emerged as the preferred procedure for permanent tooth loss treatment and replaced the use of dentures. Biocompatibility and low cost of titanium dental implants make it the most often-used choice. Titanium is a bioinert substance that has little to no negative effects on the tissue it is adhered [4,5,6]. Despite all their usefulness, dental implants can sometimes cause infection. Improper implant placement or osseointegration failure can trigger the host inflammatory response and lead to the development of peri-implantitis (PI), which has become a growing concern in dentistry because of the lack of effective treatment strategies [7].

Generally, PI was defined as an inflammatory process affecting both soft and hard tissues surrounding an osseointegrated implant, associated with suppuration or bleeding after gentle probing, resulting in quick loss of supporting bone [8]. PI can limit dental implants’ clinical success and impose health and financial burdens on patients [9,10]. There are multiple causes of PI depending on the type of implant used and the overall health status of the patient such as poor oral hygiene, smoking, diabetes, history of periodontal disease, and previous implant loss [11,12]. According to Papi et al. [13], a higher risk of PI was observed in people with hyperglycemia.

Metabolic syndrome is a wide spectrum of health disorders, including hyperglycemia, dyslipidemia, visceral obesity, rheumatoid arthritis hypertension, etc. [14]. There is no doubt that the presence of metabolic disorders while studying implant engraftment in animals and patients complicates the process of survival of implants and increases the risk of PI relative to the healthy population [14,15,16]. In this regard, the frequency of PI increases strongly in older patients [17]. High life expectancy and reliability of modern implant dentistry are all factors that lead to the increased percentage of dental implants in elderly patients and risk an increase in the number of side effects, i.e., PI [13].

The inflammatory reactions in PI affect the surrounding tissues of the implant, where the high levels of pro-inflammatory cytokines such as interleukin 1beta (IL-1β) and tumor necrosis factor-alpha (TNF-α) promote bone resorption with subsequent adverse health consequences [18]. The colonized bacteria and their biofilms formation on the implants are the significant causes of peri-implant tissue inflammation due to their interactions with the host immune system. *Streptococcus sanguinis*, *Streptococcus mitis*, *Streptococcus oralis* and actinomyces are some common bacterial species that colonize at an early stage of biofilm formation while *Porphyromonas gingivalis, Treponema denticola* and *Tannerella forsythia* are some late colonizers [19,20,21]. With the recent increasing practice of dental implants, there are parallel increases in dental implant rejection and PI cases in dentistry practices. PI therapies using biomaterials such as fibers, gels, beads, and regeneration membranes to deliver antibiotics have been effectively applied in recent years.

The crucial mediators for PI are local tissue inflammation and increased oxidative stress [22]. These mediators are also the common determinations in most systemic metabolic conditions, such as type 2 diabetes mellitus and dyslipidemia [23]. As it is known, hormones coordinate different physiological processes in the body, including various metabolic conditions, growth, and development [24]. There is no doubt that bone physiology and the repair process for implant osseointegration strongly depend on the body’s hormonal status. Martin et al. [25] conducted the transcriptome-wide gene expression analysis and found the mechanisms of upregulation of genes in the endosomal-lysosomal and oxidative stress pathway in PI. They suggested that a crucial role in PI could be the receptor-driven responses to extracellular signals, such as implant-derived titanium particles. Through the transcriptome analysis, Cho et al. [23] found that smoking differentially affected PI and periodontitis (PE) in terms of host-defense mechanism impairment.

Several systemic metabolic diseases reduce the long-term success rate of dental implants. A 16-year follow-up study reported the success rate at only 82.9%, with around 5–8% of the failed osseointegration from PI [5]. Several reports indicate associations between chronic local dental inflammation, such as PE, and the increased risks of many systemic conditions, including metabolic diseases, cardiovascular diseases, cerebrovascular diseases, and neurodegenerative disorders [23,26,27,28,29]. The incidence of peri-implantitis patients with chronic PE is 4–5 times greater than those without a burdened periodontal history [9,30].

**Figure 1:**
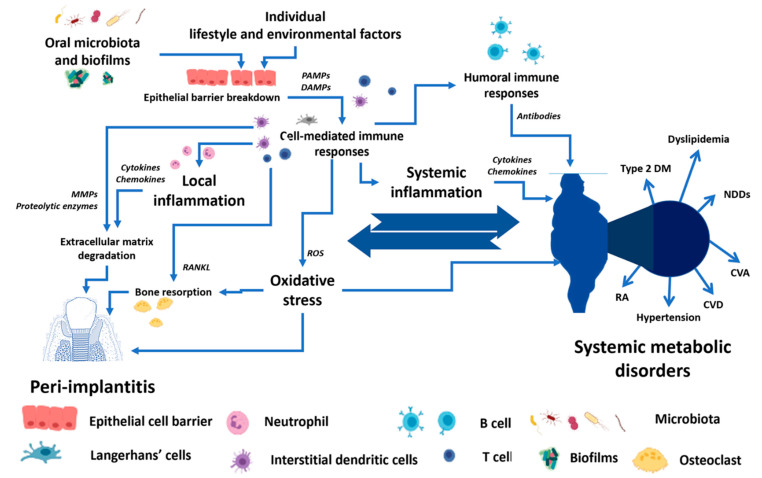
The conceptual framework of the bi-directional relationship between peri-implantitis and systemic metabolic disorders. PAMPs—Pathogen-associated molecular patterns, DAMPs—Damage-associated molecular patterns, MMPs—Matrix metalloproteinases, RANKL—Receptor activator of nuclear factor-κB ligand, ROS—Reactive oxygen species, Type 2 DM—Type 2 diabetes mellitus, NDDS—Neurodegenerative disorders, CVA—Cerebrovascular accident, CVD—Cardiovascular diseases, RA—Rheumatoid arthritis.

There are bi-directional relationships between local dental disease and systemic metabolic disorders, as shown in Figure 1. These relationships could be the crucial determinant for the success of the dental implant procedure and PI treatment. While planning and treating the local pathological oral conditions are necessary, the consideration and proper management of underlying metabolic disorders could determine the practice outcomes [5,22,31,32,33,34]. The present narrative review describes several metabolic conditions and PI.

## 2. Association between Periodontitis and Peri-Implantitis

With rising dental problems, dental implants have increased considerably in recent times. Thus, the number of individuals suffering from PI has also increased. It has been observed that individuals suffering from PE which is gum disease and affects soft tissues show a greater risk of developing PI. In other words, PE is one of the major risk factors for developing PI [35]. Interestingly, the association between PE and PI is inconsistent. For instance, some studies show a direct association between the history of PE and the development of PI in later life [36,37].

However, other studies have shown that periodontally compromised patients are more prone to PI and implant loss [38,39]. Studies have demonstrated that oral microbiota (microorganisms residing in the oral cavity collectively known as oral microbiota) associated with PE is involved in the infections related to PI, indicating a common link between PE and PI. According to Norowski and Bumgardne [9], some pathogenic species of microorganisms associated with periodontitis, such as *Aggregatibacter actinomycetemcomitans, Peptostreptococcus micros, Fusobacterium* spp, *Porphyromonas gingivalis*, etc., are also closely associated with PI. It should be mentioned that PI is regarded as a more polymicrobial disease compared to PE [40,41].

Another reason for the association between PE and PI is an observation that dental implants cause more bone loss in individuals affected with PE [35,42]. Moreover, the microbiota associated with both disorders is similar to *Treponema denticola*, *Tanarella forsythensi*, and *Prevotella intermedia*, the common bacterial species in both dental disorders. However, recent studies have shown that the core microbiota associated with PE and PI is not necessarily the same, and a distinct microbial environment is present in both diseases [35,42,43]. It is important to highlight that both PE and PI show different features at the histological level. For example, in a study by Carcuac et al., 40 patients with PE and 40 patients with PI were recruited to understand the histological and immunological differences. It was observed that PI lesions were larger in size, twice in comparison with PE, and contained a higher number and density of key immune system cells positive for cluster of differentiation 138 (CD138), CD68-, and myeloperoxidase (MPO) markers. Moreover, the infiltration of the immune system cells is more pronounced in PI than in PE. According to Yang et al. [44] and Avdeev et al. [45], modulating the proper immune microenvironment could promote periodontal regeneration.

The difference between PE and PI was also observed in the type of immune system cells infiltrated in the dental tissue. In PE, the primary infiltrating cells were lymphocytes and plasma cells, while in PI, inflammatory macrophages and neutrophils were the dominant cells [46]. The bone morphogenetic protein (BMP)/retinoic acid inducible neural specific 3 (*BRINP3)* gene has been linked with an aggressive form of PE. However, a recent study by Casado et al. has established that PI is also associated with *BRINP3* polymorphic variant rs1342913, but the association is independent of PE. Thus, based on the available scientific evidence, it can be concluded that PE is a risk factor for PI, but both are not identical at histological and immunological levels [47].

Recently, Martin et al. [25] concluded that three core clinical features suggest that PI is very dissimilar to PE: (1) the bone destruction in PI is more significant than observed in PE; (2) clinical studies reported implant-derived titanium particles as being substantially increased in the peri-implant microenvironment in PI versus health; (3) mechanical and antimicrobial interventions that are very effective against PE have limited influence against PI which is regarded to a leading role of persistent titanium particles in PI. For instance, Safioti et al. [48] monitored the increased levels of dissolved titanium in PI.

## 3. Microbiota

There is no doubt that PI has been associated with the formation of dental plaque on implants [49,50]. Once inserted and fixed into the oral cavity, the surface of dental implants is colonized by the microorganisms within 30 min of the implantation. The colonization of bacterial species promotes the formation of biofilms on the implant surface [35]. In case dental implants are strongly influenced by colonization with pathogenic acid-producing bacterial strains or Gram-negative anaerobes, inflammatory reactions develop, which can result in PI [9]. 

The oral cavity is home to a range of microbial species, and an estimated 700 bacterial species and 25,000 phylotypes are found in the oral cavity. It has been observed that the bacterial composition of the biofilms developed on the dental implant is influenced by the microbial communities present on the neighboring teeth. A common observation is that PE and PI are similar in oral microflora [35]. Interestingly, the microbiota species in healthy dental implants were more complex and diverse compared to microbial communities in PI, indicating that beneficial implants harbor a stable microbiota. However, other studies have shown a higher microbial diversity in PI patients. Some of the most common bacterial species associated with PI are *Tannerella, Treponema, Prevotella*, and *Bacteroides* [35]. 

Moreover, the diversity of bacterial species increases with the progression of the disease. Studies have shown that other bacterial species associated with PI are asaccharolytic anaerobic Gram-positive rods and oxidized graphene nanoribbons apart from periodontopathic bacteria. In addition, *Campylobacter rectus* and *Aggregatibacter actinomycetemcomitans* have also been identified in PI [35]. In another observation, it has been shown that *Streptococcus* and *Actinobacillus* were more common in healthy subjects, while a higher presence of *Porphyromonasand prevotella* has been reported in PI subjects. Another bacterial phylum, *Synergistetes* was also found in PI subjects [51]. 

Moreover, normal implants and disease implants harbor different microbiological ecosystems. Studies have shown that microbial communities present in a PI site are a heterogeneous mixture of Gram-positive and Gram-negative rods, enteric rods, and *Staphylococcus aureus.* The microbial communities found in the PI predominantly make red-disease-and-orange color complexes. For example, the red color complex is formed by *Treponema denticiola, Porphyromonas gingivalis,* and *Tannerella forsythia,* while the orange color complex is formed by *Prevotella intermedia* and *Fusobacterium nucleatum. Candida albicans,* a fungus, have shown a higher tendency to attach to titanium dental implants under in vitro studies [52]. Romeo et al. [53] found that such bacterial species as *Staphylococcus aureus*, *Pseudomonas aeruginosa, Enterobacteriaceae* spp., and *Candida albicans* were also identified around implants. Still, they mainly reflect opportunistic plaque colonization according to antibiotic treatments.

Another important aspect of bacterial colonization on dental implants is the formation of biofilms. Biofilms play a very important role in the adhesion of various bacterial species on the implant surface and provide a suitable ground for bacterial adhesion. Biofilms are so important that recent studies have proposed inhibitors of biofilm formation as a possible therapeutic option to prevent bacterial adhesion on implant surfaces [54].

The microbial communities of the oral cavity are quite dynamic due to creating symbiotic complexes of bacteria, which can change quickly regarding antimicrobial treatment, which makes the effective combating of PI a difficult task [9]. The transfer of antibiotic-resistant genes between different microbial species in biofilms contributes to developing drug-resistant bacteria [55]. The experimental evidence of biomaterial therapies using gels, fibers, beads, and tissue regeneration membranes to deliver antibiotics have been effectively used in the coping PI. Nevertheless, their clinical efficacy is not well documented [9].

## 4. Oxidative Stress

Reactive oxygen species are produced in the cells due to normal metabolic processes. However, they potentially damage various cellular macromolecules, such as nucleic acids, proteins, and lipids. Studies have demonstrated that increased oxidative stress is an important factor in dental disorders such as periodontitis, and affected individuals display an imbalance in the defense to fight oxidative stress leading to tissue damage in periodontitis. Although PI is a common issue related to dental implants, the underlying mechanism is not yet discovered completely [56]. A recent study has demonstrated that dental implants release titanium nanoparticles in the surrounding, which subsequently promotes tissue inflammation, oxidative stress, and bone damage [57]. The study used mesenchymal stem cells and fibroblasts and exposed them to titanium nanoparticles. It was demonstrated that titanium nanoparticles decreased the cell viability time-dependent and stimulated the synthesis of reactive oxygen species. The in vivo analysis shows abnormal extracellular titanium levels and bone metabolism disorders [57]. Furthermore, a higher oxidative stress milieu also promoted neutrophil recruitment and higher expression of tissue metalloproteinase leading to the degradation of tissue collagen [57]. A study by Jazi et al. measured the levels of superoxide dismutase (SOD), total antioxidant capacity (TAC), and malondialdehyde (MDA) in the peri-implant crevicular fluid. The study concluded that the MDA, TAC, and SOD levels could not differentiate between peri-implant health and disease [56]. In another study by Song et al., SOD and glutathione peroxidase (GPx) were measured in individuals with PI and healthy implant groups. The SOD and GPx levels were significantly lower in individuals with PI than in beneficial implants indicating a lower ability of PI subjects to fight cellular oxidative stress. The study also observed higher levels of various pro-inflammatory cytokines in PI subjects [58].

Increased level of cellular reactive oxygen species also induces various glycoxidation reactions leading to the formation of advanced glycation end products (AGEs). Abnormal levels of AGEs promote cross-linking of cellular proteins and protein aggregation, leading to the activation of various cell signaling pathways and subsequent cell damage and apoptosis [59].

A recent case/control study has measured the levels of AGEs in PI subjects and observed that affected individuals show higher levels of AGEs and ThioBarbituric Acid Reactive Substances compared to healthy subjects. Taken together, higher levels of oxidative stress in one of the underlying causes of PI, and therapeutic strategies to reduce oxidative stress could be a potential approach to managing PI [60].

## 5. Inflammation

Inflammation is a process in which cells or tissues become inflamed due to infection caused by microorganisms. Like oxidative stress, the inflammatory process also plays a pivotal role in the etiology of PI. Landgraeber et al. [61] concluded that the failure of implants is mediated by cytokines determined by the innate immune system. It should be noted that low-grade inflammation, which accompanies chronic periodontal infections, can significantly damage the metabolic processes in the body [62].

The role of key inflammatory cytokines such as IL-1 and TNF-α in PE is well documented. Abnormal expression of these cytokines contributes to tissue destruction and alveolar bone loss [63]. Similarly, a higher expression of these cytokines has also been reported in PI [64]. Measurement of TNF-α and IL-1β in PI subjects has been explored for early diagnosis of the disease. IL-1β has been shown to differentiate between normal and PI implants [64]. It has been observed that IL-8 is also increased in PI subjects. 

Moreover, the levels of peroxisome proliferator-activated receptors gamma, an anti-inflammatory mediator in the cells, decrease in PI subjects. Another anti-inflammatory cytokine, IL-10, was also reduced in PI. A higher level of IL-1β and reduced levels of IL-10 also increased the ratio of IL-1β/ IL-10, indicating a higher inflammatory condition in PI subjects than the healthy implant sites. Neutrophil infiltration has been shown to promote inflammation in the peri-implant sites [64]. One of the major markers of neutrophil infiltration is myeloperoxidase (MPO), a peroxidase secreted by neutrophils. A recent study measured the levels of MPO in peri-implant sulcus fluid of implants (PISF) and gingival crevicular fluid (GCF) in both healthy and diseased states. It was observed that MPO activity was significantly higher in PISF with inflamed sites than in non-inflamed areas. MPO can be used as a diagnostic marker to identify the disease condition [65]. In another study, total salivary malondialdehyde and MPO levels were higher in PI subjects than in healthy implant sites [66].

## 6. Bone Resorption

Expression of various inflammatory molecules and matrix metalloproteinases in the lesions surroundings the dental implants can change the expression profile of various mediators involved in bone resorption/remodeling. Moreover, these inflammatory mediators can also attract the chemotaxis of osteoclast cells [64]. A study by Arikan et al. showed that individuals with PI displayed higher levels of C-telopeptide pyridinoline crosslinks of type I collagen (ITCP) in their peri-implant fluid than normal [67]. It is important to mention that ITCP is an important marker for various osteolytic bone diseases such as osteoarthritis and osteoporosis. Thus, a higher level of ITCP in PI subjects shows increased bone destruction [68].

Moreover, Arikan et al. also found a lower concentration of osteoprotegerin (OPG) in the PI group than in the healthy group. OPG is an inhibitor of osteoclast cells, and its lower levels have been associated with bone disorders such as osteoporosis. Thus, higher ITCP and lower OPG are important markers for bone loss in PI [69].

It has been shown that PI is associated with reduced levels of secreted phosphoprotein 1, bone gamma-carboxyglutamate protein, and collagen type 9 alpha 1 chain, key markers for bone matrix [70]. Moreover, higher expression of fibrocyte markers has been observed in PI. This is important because fibroblasts have been implicated in the degradation of the matrix and hence aggravate the symptoms of PI. Another important marker for bone loss is bone morphogenetic protein 7 (BMP-7), which is down-regulated in the inflamed bones of PI [64,70].

## 7. Association with Systemic Conditions

PI is associated with certain systemic conditions which are responsible for aggravating the symptoms of PI. Smoking and diabetes were regarded as the most universally accepted risk factors for developing PI by Kormas et al. [12]. Some major systemic conditions and their association with PI are presented below.

### 7.1. Obesity

Obesity is a lifestyle-related disorder, and the incidences of obesity have increased several folds in recent decades due to an unhealthy lifestyle and intake of energy-dense foods. One of the characteristic features of obesity is the presence of a chronic inflammatory milieu in the system called “Chronic-low grade inflammation” [71]. As discussed above, inflammation is also an underlying cause of PI. Moreover, the severity of PI is associated with obesity. For instance, a recent study by Vohra et al. measured the levels of pro-inflammatory cytokine C-reactive protein (CRP) in obese individuals with PI. The study reported that inflammatory parameters, such as marginal bone loss and bleeding on probing, were significantly higher in severe forms of obesity [72]. The same trend was observed for CRP levels. In another clinical study, levels of inflammatory cytokine, IL-6, and IL-1β were measured in obese and non-obese individuals with PI. It was observed that obese individuals had a higher salivary concentration of these inflammatory cytokines than non-obese subjects. Moreover, obese individuals also showed higher bleeding on probing and probing depth than non-obese subjects. This study shows that obese subjects are prone to developing peri-implant inflammation compared to non-obese subjects [73].

### 7.2. Diabetes

Diabetes mellitus is a disorder of abnormal glucose metabolism and leads to hyperglycemia. Diabetes is associated with several health complications, such as micro-and macrovascular disorders, thus adversely affecting normal body homeostasis. It has been observed that people with diabetes show delayed wound healing, reduced ability to fight infections, tooth loss, and periodontitis [74,75]. A study by Aguilar-Salvatierra has found an association between diabetes and PI. The glycated hemoglobin levels were directly correlated with inflammation in PI subjects [76].

A systemic review by Naujokat et al. has observed that individuals with diabetes can safely opt for dental implants. There is no significant difference in the survival rate of dental implants between diabetics and non-diabetics for up to 6 years. Still, a reduction in dental implant survival rate was observed in the long term (20 years). Moreover, individuals with poor diabetes control require a higher time for osseointegration. However, the difference seems irrelevant one year after implantation, even in individuals with poorly controlled glycated hemoglobin A1c (HbA1c) [77]. Another observation was that inflammation associated with PI is higher in people with diabetes only in the long run, and initial years seem normal in people with diabetes. The study concluded that maintaining good control of diabetes leads to better integration of dental implants and certainly reduces the risk of infection in the implant. In other words, managing glucose levels improves the chances of implant integration and inflammation [77]. Thus, measurement of HbA1c and prescription of better antidiabetic drugs to improve hyperglycemia pave the way for higher chances of implant survival. As an additional precaution, supportive therapies such as antibiotics and chlorhexidine are recommended to reduce the chances of infection [77]. Experimental studies conducted by De Molon et al. [78] revealed that diabetes mellitus worsened the bone healing around implants. Insulin therapy effectively prevented the occurrence of bone abnormalities in diabetic animals and improved osseointegration [78].

### 7.3. *Rheumatoid Arthritis*

Rheumatoid arthritis (RA) is an autoimmune disorder and affects up to 1% of individuals in developed nations. Some studies have observed the effect of RA on the success rate of dental implants [79]. It was reported that individuals with RA show a success rate for dental implants ranging from 93.8–96.1% [80]. Studies with the role of RA in PI have not found any significant difference between implant success rate and RA. However, Krennmair et al. reported that RA-affected patients displayed higher bleeding on probing and crestal bone resorption [81].

Dyslipidemia is one of the consequences of abnormal lipid metabolism in the body. One of the major factors for developing dyslipidemia is the intake of a high-fat diet, which leads to abnormal levels of total cholesterol and triglycerides. Dyslipidemia is related to cardiovascular disorders and osteoporosis. Interestingly, dyslipidemia also adversely affects bone density and strength, and lipid-lowering drugs such as statins positively influence bone metabolism. Since bone density and strength are directly correlated with the osseointegration of dental implants, abnormal lipid levels can hamper the normal integration of dental implants. In a recent study in C57BL/6J male mice, Keuroghlian et al. evaluated the effects of hyperlipidemia on dental implants [82]. Dyslipidemia was induced in mice by feeding them a high-fat diet for 12 weeks, after which a titanium-made dental implant was placed in the proximal metaphysis of the femur. The study reported that HFD-fed mice showed significantly higher implant loss and reduced interaction between the bone and the implant, indicating reduced osseointegration. The study established that dyslipidemia can significantly reduce outcomes in dental implants. In a similar observation by Tekin et al., hyperlipidemia reduced bone-to-implant contact, consequently reducing peri-implant bone regeneration [83].

Based on the above-cited studies, it can be safely concluded that the successful integration of dental implants depends on various systemic disorders and health issues. These systemic issues can adversely affect bone integration and implant success by increasing the inflammatory milieu in the surroundings of the implant, lowering the bone strength and density to prevent normal osseointegration, increasing oxidative stress, and allowing the overexpression of various inflammatory mediators. Thus, it can be concluded that managing various systemic disorders such as obesity, type 2 diabetes mellitus, dyslipidemia, and RA can increase the implant success rate and reduce the chances of PI. However, the research in this direction needs to be improved, and a better understanding is still required.

Summarizing the abovementioned data, it is worth noting that the individual lifestyle and environmental factors, together with the breakdown of the epithelial cell barrier, allow the invasion of oral microbiota and their biofilms, which activate the cell-mediated immune responses. The pro-inflammatory cytokines and increased oxidative stress in local tissue lead to extracellular matrix destruction and bone resorption in PI. The un- or undertreated PI can escalate into systemic metabolic conditions through the common mediators of systemic inflammation and oxidative stress. However, the underlying systemic metabolic disorders can increase the risk of PI (see Figure 1).

## 8. Preventing and Management of Peri-Implantitis Regarding Different Metabolic Conditions in Patients

After diagnosis of PI, many therapeutic approaches are present for saving the implant and avoiding its removal [84]. These strategies follow such general stages as mechanical removal of the biofilm from the peri-implant pocket, antiseptic treatment, antibiotic therapy to eliminate pathogenic bacteria in the surrounding tissues, and osseo-reintegration if necessary. Yi, M. et al. [24] demonstrated that systemic hormone intake through various drug-delivery systems could effectively regulate the osseointegration of dental implants and whole metabolism. The listed approaches, except pharmacotherapy involving antiseptics and antibiotics, are beyond the scope of this chapter.

The clinical studies showed that local antibiotic administration effectively reduced bleeding on probing and peri-implant probing depth in patients affected by PI [11]. If the titanium implant’s surface is loaded with antibiotics, it could target implant-associated polymicrobial infection [85]. Recently, researchers and clinicians have been developing nano-modified titanium implants with antimicrobial properties [86]. Zhang et al. [87] found that the drug-delivery abutment used in the in vitro and in vivo experiments effectively delivered minocycline hydrochloride into peri-implant tissues and, thus, prevented or even treated peri-implant infections. On the other side, Polymeri et al. [88] found that administering systemic antibiotics (amoxicillin and metronidazole) in patients with PI did not show statistically significant healing effects. They could potentially increase the problem of antibiotic resistance. Similar results were obtained by van Winkelhoff, A.J. [89]. Similarly, Bizzarro et al. [90] found that systemic antimicrobials in periodontitis treatment have negative effects on the parameters of metabolic syndrome. Research conducted by D’Aiuto et al. [91] in the USA, established significant relationships between metabolic disorders and severe PE.

As the main drawback of metallic nanoparticle films, which reduce the growth of oral pathogenic bacteria, are the accumulative effect and potential toxicity of the metals over time, using biomaterials as carriers of antimicrobial drugs has attracted substantial attention for application as coatings on implant devices [41]. Wang et al. [92] reported that mechanical debridement with antimicrobial photodynamic therapy was effective in treating peri-implant diseases. It may change oral microbiota composition by increasing the abundance of beneficial microorganisms and decreasing harmful ones. Antimicrobial photodynamic therapy was considered by Zhao et al. [93] as an alternative to antibiotics in the treatment of PI and periodontitis.

The controlled release of antibiotics and antiseptics supplies many devices such as polymeric fibers, gels, chips, or microcapsules [9,94]. These devices are intended to keep the necessary concentration of antimicrobial agents (chlorhexidine, tetracycline, minocycline, doxycycline, metronidazole, etc.) in the periodontal pocket locally for an extended period [95]. Recently, the polymeric chlorhexidine gluconate coating of the internal chamber of the implant demonstrated the ability to control bacterial loading [49].

Preventing PI was recognized as a justified strategy concerning the health and cost of patients [9]. The anti-bio adhesion coatings of implant surfaces, antibiotic-releasing coatings antibiotic-releasing as well as covalent modification of surfaces, and the use of photocatalytic and Ag/Zn modified surfaces were regarded as the effective approaches to prevent PI [9,96,97,98]. As it is known, biofilm control is crucial for preventing caries and periodontal diseases. It was found that as an artificial sweetener, xylitol can inhibit dental biofilm formation by decreasing bacterial β-glucosidase in human saliva [99,100]. Xylitol can also efficiently stimulate the immune system, as well as improve bone and lipid metabolism, having a positive impact on metabolic syndrome [99].

Many natural compounds could be effectively used in combating metabolic syndrome and, thus, reduce the risk of PI development [101]. For instance, diterpene totarol was applied by Xu et al. [102] as a natural antibacterial coating on dental implants to prevent PI. The probiotic bacterial strain of *Lactobacillus plantarum* produced the bacteriostatic peptide called bacteriocin. The probiotic has advantages compared to antibiotics, such as no resistance from periodontal pathogens and easy degradation in the human gut [103]. A randomized clinical study investigated the effects of a probiotic tablet containing *Lactobacillus reuteri* in 30 peri-implantitis patients. Results of this demonstrated that after administration, bacterial growth decreased but again increase in the number of bacteria was observed after some time. Thus, probiotics can prevent inflammation instead of improving oral microbiota in PI [104]. Currently, very limited information is present to analyze and validate the rationale and effectiveness of probiotics as adjuncts in peri-implantitis therapy. The available data are based on periodontitis management objectives and strategies. As it was demonstrated recently, periodontitis is connected with thyroid dysfunction and therefore these data should be taken into account in its treatment [105]. Some studies have reported differing and contradictory findings [106,107,108,109,110].

Curcumin-loaded polymeric nanoparticles were effectively used for antimicrobial photodynamic chemotherapy in PI [111].

It should be noted that nano-formulations with natural polyphenol curcumin are widely applied in the prevention and treatment of inflammation-related diseases due to their prominent antioxidant properties [112]. Propolis, a resinous product collected by bees from phytoexudates to seal cracks and holes in their hives, was regarded as a suitable bio-substance to be incorporated into dental biomaterials due to its significant antimicrobial, immuno-modulating/anti-inflammatory properties [113,114]. Propolis is a cost-effective and safe product with minimal adverse effects [113]. Somsanith et al. [115] demonstrated in vivo the enhancement of osseointegration with propolis-loaded titanium nanotubes for dental implants. Martorano-Fernandes et al. [116] revealed the inhibitory effect of Brazilian samples of red propolis on developing *Candida* biofilms on titanium surfaces.

Table 1 represents some of the current approaches used to manage peri-implantitis.

## 9. Conclusions

Dental implants have become routine dentistry management for tooth-related conditions such as tooth loss. There is an increasing incidence of PI following dental implants, which induce abnormal inflammation in the surrounding tissues, bone loss, or even implant failure. Local peri-implant tissue health is crucial for the successful integration of implants. The healthy epithelial barrier helps to prevent the entry of microbes and their biofilms and lessen the microbe-immune interactions with subsequent inflammation and oxidative stress. Interestingly, patients with underlying systemic metabolic disorders, such as obesity, dyslipidemia, diabetes mellitus, or rheumatoid arthritis, have an increased risk of PI. Pre-procedure planning should consider both local and systemic individual factors to increase the chances of success. During the PI treatment, proper management of systemic metabolic conditions and smoking cessation are critical measures that need to be considered along with the local dental treatment.

## Figures and Tables

**Table 1:** Management strategies to control peri-implantitis.

Strategy	Effect	References
Administration of antibiotics (such as minocycline, doxycycline, tetracycline, and metronidazole)	Reduction in peri-implant probing depths and bleeding on probing without causing any negative side effects	[11]
Titanium surface loaded with poly-antimicrobials	Manage polymicrobial biofilms caused by implants	[85]
Nanoengineered titanium dental implants	Inhibit the growth of biofilm-forming bacterial species in dental implant surroundings	[86]
Mechanical debridement (MD) + antimicrobial photodynamic therapy (aPDT)	Improves peri-implantitis by increasing the population of beneficial bacteria and decreasing the harmful bacteria	[92]
aPDT	Higher antibacterial efficiency in PI	[93]
Anti-bio adhesion coatings of implant surfaces	Reduce adhesion of harmful microbes to the dental implant surface	[117]
Antibiotic-releasing coatings on implant surface	Reduction in bacterial infections in dental implants	[118]
Covalent modification of implant surfaces	Improved osseointegration of titanium dental implants	[119]
Ag/Zn modified implant surfaces	Reduction in bacterial infections in dental implants	[120]

## Data Availability

Not applicable.
